# Disposable microfluidic micromixers for effective capture of *Cryptosporidium parvum* oocysts from water samples

**DOI:** 10.1186/s13036-018-0095-6

**Published:** 2018-03-27

**Authors:** L. Diéguez, M. Winter, S. Molan, P. Monis, B. King, B. Thierry

**Affiliations:** 1 Future Industries Institute and ARC Centre of Excellence in Convergent Bio and Nano Science and Technology, University of South Australia, Mawson Lakes Campus, Mawson Lakes, South Australia Australia; 2 International Iberian Nanotechnology Laboratory, Braga, Portugal; 3 South Australian Water Corporation, Adelaide, SA Australia

**Keywords:** *Cryptosporidium parvum* oocysts, Disposable microfluidic micromixers, Imaging flow cytometry, Water quality, Immunocytochemistry, Fluorescence microscopy

## Abstract

**Background:**

Protecting drinking water supplies from pathogens such as *Cryptosporidium parvum* is a major concern for water utilities worldwide. The sensitivity and specificity of current detection methods are largely determined by the effectiveness of the concentration and separation methods used. The purpose of this study is to develop micromixers able to specifically isolate and concentrate *Cryptosporidium*, while allowing in situ analysis.

**Results:**

In this study, disposable microfluidic micromixers were fabricated to effectively isolate *Cryptosporidium parvum* oocysts from water samples, while allowing direct observation and enabling quantification of oocysts captured in the device using high quality immunofluorescence microscopy. In parallel, quantitative analysis of the capture yield was carried out by analyzing the waste from the microfluidics outlet with an Imaging Flow Cytometer. At the optimal flow rate, capture efficiencies up to 96% were achieved in spiked samples.

**Conclusions:**

Scaled microfluidic isolation and detection of *Cryptosporidium parvum* will provide a faster and more efficient detection method for *Cryptosporidium* compared to other available laboratory-scale technologies.

## Background

*Cryptosporidium* is a highly resistant protozoan commonly encountered in surface waters. Although *Cryptosporidium* infections are self-limiting in healthy individuals, the consequences can be far more serious in infants and young children causing moderate to severe diarrhea [1]. *Cryptosporidium* outbreaks are often linked to treatment failures or treatment deficiencies at water treatment plants, allowing contamination of drinking water. Risk management of source waters requires cost effective, rapid and efficient monitoring of *Cryptosporidium* [2]. Procedures for *Cryptosporidium* detection typically includes collection of a large volume of water sample (10 - 1000 L), followed by concentration using various techniques including filtration and centrifugation to obtain a concentrated sample. The standardized procedure EPA 1623 describes a complete isolation/detection protocol based on filtration, elution from the filter and centrifugation to obtain a concentrated sample. The recovered sample then undergoes immunomagnetic separation to isolate oocysts from debris. Isolated oocysts are stained with specific fluorescent antibodies and nuclear stain for detection by immunofluorescence and microscopy. This method has demonstrated a recovery yield of 61% in spiked pure water, 48.8% in spiked filter tap samples and 19.5% in raw sources, with a limit of detection of approximately 10 oocysts/L [3]. The final steps in the EPA 1623 method require skilled technicians and are time- and resource-consuming. While immunofluorescence microscopy is the gold standard for the detection of the oocysts, other antigen based detection methods including ELISA (enzyme-linked immunosorbent assay) and immunochromatographic assays are also commercially available [4].

A number of alternative approaches have been reported to detect waterborne pathogens [5], including those based on Surface Plasmon Resonance [6], nucleic acid detection [7–11], immunocantilevers [12], Surface Enhanced Raman Scattering [13], dielectrophoresis [14] and impedance spectroscopy [15]. But all detection methods rely on efficient concentration of large volume of water sample and isolation of the pathogen. A number of microfabricated filters [16–18] have been described and yielded oocysts capture efficiencies as high as 97% in spiked pure water samples. Microfluidic methods have also been actively investigated in recent years for oocysts detection due to their excellent reliability and efficiency. Owing to the inability of microfluidic systems to deal with very large volumes, these approaches have typically focused on treating samples that have already been subjected to the initial concentration step. Microfluidic filters achieved an efficiency of 86% when capturing *Cryptosporidium* oocysts from spiked concentrated water samples [19]. Inertial microfluidics has also been applied with some success to enrich water and food pathogens, yielding 100% efficiency sorting *Cryptosporidium* from concentrated water [20] and 68.4% when recovering *Giardia* from food samples [21]. The Nano-DEP enrichment system provided a 10 times concentration of the raw sample [13]. These microfluidic systems rely on physical filtration or separation of oocysts from a pre-concentrated sample to deliver the sample for the final detection step. The pre-concentrated samples still need to be further processed to be analyzed using for example fluorescence microscopy. In addition, approaches based on physical features lack specificity in comparison to standard immunomagnetic separation. With this in mind, the integration of immunomagnetic separation with microfluidics has been advanced [22]. In addition, good recovery yield using microfabricated microwells functionalized with antibodies has also been reported, although the static nature of this approach limits its application to only a small volume of sample [23]. Recently, McGrath et al. reported on the interesting concept of high throughput Microfluidic Impedance Cytometry (MIC) for rapid enumeration and identification of different types of *Cryptosporidium* spiked in saline buffer and achieved over 92% accuracy in discriminating *Cryptosporidium parvum*, *Cryptosporidium muris* and *Giardia lamblia* [24].

Microfluidic devices bioconjugated with specific molecular probes have been used with remarkable success in the isolation of circulating tumor cells from the blood of cancer patients [25]. To perform efficient immunocapture of cells in functionalized microfluidic devices, it is necessary to maximize the surface to volume ratio and to create appropriate mixing to optimize the chances of the target cells coming into contact with the immunoconjugated surface. To our knowledge, the application of a biofunctionalised micromixer for the immunospecific capture of *Cryptosporidium* has not been investigated thus far. Towards simplifying the workflow of *Cryptosporidium* oocysts detection from a pre-concentrated water sample with increasing accuracy, the aim of this work is to evaluate a streamlined microfluidic technology for its ability to specifically isolate *Cryptosporidium* oocysts from concentrated water samples while simultaneously allowing for high quality immunofluorescence observation. In this way, oocysts present in pre-concentrated samples (using any of the above mentioned approaches) can be specifically isolated and quantified in a single system. A positive enrichment strategy has been used due to sample/technical specificities including multiple contamination source in water samples and the existence of an antibody that binds the target oocysts with high specificity and affinity. For this purpose, disposable microfluidic devices were fabricated and functionalized with an antibody (Cry 104) specific against antigens expressed on the oocyst surface. The microfluidic device consists of an array of 25 μm thin micromixers designed to enhance the surface interactions between the *Cryptosporidium* and the channel walls. Once isolated within the micromixer, the oocysts can be readily stained using standard fluorescent tags and observed in situ under fluorescence microscopy, providing a simpler alternative to the current method based on immunomagnetic separation. Under optimal conditions, a recovery yield of up to 96% could be obtained for *Cryptosporidium* oocysts spiked in water.

The design of this microfluidic device enhances mixing and hence binding of the *Cryptosporodium* with its many parallel channels which not only increase the area for binding but also provides some redundancy for blockage as would be expected with environmental water.

## Methods

The micromixers were fabricated in SU8 on a silicon wafer. The design consists of an array of 25 μm high microchannels favoring chaotic mixing. Standard soft lithography was used to produce disposable microfluidic devices, sealed with oxygen plasma. Using a silane-based functionalization strategy, antibodies were immobilized on the channel surface. Known numbers of *Cryptosporidium* were spiked in saline buffer and introduced through the microfluidic devices at different flow rates. The oocysts captured in the device were washed, fixed, permeabilized and stained prior to microscope examination. The waste solution recovered from the devices was also analyzed with an Imaging Flow Cytometer Image Stream X (ISX, AMNIS, Seattle, WA, USA).

### Materials

Phosphate buffered saline (PBS), (3-aminopropyl) trimethoxysilane (APTMS), trichloro 1,1,2,2-perfluorooctyl-silane, bovine serum albumin (BSA), fetal bovine serum (FBS), glutaraldehyde, formaldehyde, Triton™ X-100, and 4′,6-Diamidino-2-phenylindole dihydrochloride (DAPI) were purchased from Sigma Aldrich (USA). γ-irradiated *Cryptosporidium parvum* were kindly donated from SA Water (Australia). Specific monoclonal antibody Cry104 was obtained from BTF Biomerieux (Australia). A FITC goat anti mouse IgG secondary antibody was purchased from Sigma Aldrich (USA). Polydimethylsiloxane (PDMS) elastomer SYLGARD 184 was obtained from Dow Corning (USA) and SU-8 10 photoresist was purchased from MicroChem (USA). All other chemicals were analytical grade. Silicon wafers with a 3″ diameter were obtained from Micro Materials & Research Cons. Pty Ltd (Australia), and the syringe pumps KDS-212-CE and KDS-210 used in this study were purchased from KD Scientific.

### Fabrication of microfluidic devices

Standard photolithography was used to fabricate chaotic mixing silicon masters for PDMS molding, as previously described [26]. Briefly, silicon substrates were cleaned with acetone in a sonic bath for 5 min and then with isopropanol for 5 min. After rinsing with isopropanol, substrates were dried with a nitrogen gun and activated with oxygen plasma for 10 min. The negative photoresist, SU-8 10, was then spin-coated onto the wafer. The SU-8 was then patterned using a film photomask (JD tools) with a UV dose of 225 mJ/cm^2^ and post-baked at 65 °C for 1 min and at 95 °C for 2 min. SU-8 was then developed during 2 min to form a template and hard-baked with a ramping temperature from 65 °C to 200 °C.

The template was hydrophobized submitting it to a trichloro 1,1,2,2-perfluorooctyl-silane vapor in a desiccator for 1 h at 80 °C, and covered with liquid PDMS (the PDMS prepolymer was mixed with the cross-linker at a 10:1 ratio and degassed). PDMS was then degassed, cured at 80 °C for 1 h and unmolded from the silicon master. Inlet and outlet were punched in the PDMS replica that was then irreversibly sealed against a clean glass slide upon treatment in oxygen plasma at low power for 15 s.

### Functionalization of the microfluidic devices

After attaching tubing to the inlet and outlet ports, the micromixers were connected to a syringe pump and filled with ethanol at a flow rate of 100 μl/min. Once the devices were stabilized, 2% APTMS in ethanol was withdrawn into the device for 30 min and rinsed with ethanol for 10 min. The buffer solution was then changed with MilliQ water and stabilized for 10 min prior to withdrawal of 1% glutaraldehyde in water for another 30 min and rinsing in ultrapure water for 10 min. PBS was then withdrawn into the device and equilibrated for 10 min just before introducing 200 μl of 50 μg/ml Cry104 in PBS that was left to react overnight at 4 °C. Unreacted antibodies were rinsed with PBS and the surface blocked with 2% BSA in PBS. All the functionalization steps were done at the same flow rate. Control devices were functionalized following the same protocol, but without the antibody conjugation.

### Capture of *Cryptosporidium parvum* oocysts

To optimize the parameters for the isolation of *Cryptosporidium*, small volumes (50 μl) of high concentrated spiked samples in PBS (1.5 × 10^6^ and 1.5 × 10^4^ oocysts/ml) were injected into the functionalized micromixer at flow rates of 0.5, 2 or 5 μl/min to allow binding of the oocysts to the specific antibodies and explore the effect of flow rate. Washing of unbound oocysts was conducted at the same flow as for binding of oocysts, i.e. 0.5, 2 or 5 μl/min.

### Fluorescence microscopy studies

The microfluidic devices were examined under a Nikon Ti Eclipse inverted fluorescence microscope. The oocysts isolated in the device were fixed with 4% formaldehyde, permeabilised with 0.05% Triton X-100 and stained with 1:10,000 DAPI at 0.5, 2 or 5 μl/min (same as flow rate used for oocyte binding) to allow identification by fluorescence microscopy as a proof of concept. The presence of DAPI fluorescent bodies identified as oocysts captured inside the device was quantified in situ by imaging 50 randomly chosen low magnification fields of view in different sections of the device, using a 10× objective.

Towards demonstrating the relevance of this approach for environmental samples, the presence of *Cryptosporidium* oocysts was also confirmed using the Cry104 antibody. Briefly, after capture, the device was incubated with the Cry104 antibody (1/50) for 60 min. After washing with PBS, permeabilisation and blocking was performed with 0.05% Triton X-100 and 2% BSA for 10 min. The devices were then incubated with the FITC goat anti mouse IgG secondary antibody for 30 min as per the manufacturer’s instructions, before washing and imaging.

### Imaging flow cytometry studies

To quantitatively characterise the performance of the device and calculate the isolation yield of the micromixer, the input solution and the solution eluted from the device were recovered and analyzed with an Imaging Flow Cytometer (IFC) Image Stream X (AMNIS, Seattle, WA, USA). Solutions were centrifuged at 10,000 RCF for 15 min at 4 °C. The supernatants were removed and the pellets resuspended to 100 μl for Imaging Flow analysis. Since *Cryptosporidium* particles were already stained with DAPI during the microfluidic procedure, channel 1 of the flow cytometer was set to record all the DAPI stained events, with the 405 laser. Channel 4 and 6 were set for brightfield and darkfield images, respectively. In order to discriminate the oocysts from autofluorescent debris, a size classification was applied.

To confirm that the DAPI-stained bodies identified as oocysts were indeed *Cryptosporidium parvum*, the recovered sample was labelled with the specific Cry104 antibody and a goat anti mouse FITC IgG secondary antibody was used. Briefly, after blocking with 10% fetal bovine serum in PBS for 10 min on ice, the sample was incubated with the Cry104 antibody (1/50) for 60 min. After washing with PBS, 0.05% Triton X-100 and 1% FBS, the sample was incubated with the FITC secondary antibody (1/5000) for 30 min. After washing with PBS samples were run through the IFC. The FITC events were recorded in Channel 2 of the IFC with the 488 laser. Controls were performed to discriminate non-specific staining.

The capture efficiency was calculated based on eq. 1.

1 $$ \boldsymbol{Capture}\ \boldsymbol{Efficiency}\ \left(\%\right)=\frac{\left(\#\boldsymbol{oocytes}\ \boldsymbol{in}\boldsymbol{troduced}\right)-\left(\#\boldsymbol{oocytes}\ \boldsymbol{in}\ \boldsymbol{waste}\right)}{\left(\#\boldsymbol{oocytes}\ \boldsymbol{in}\boldsymbol{troduced}\right)}\times \mathbf{100} $$

### Statistical analysis

Statistical analysis was performed in Matlab (2017b, Mathworks) and Excel. Student’s *t-tests* were used for directly comparing two variables. A 1-way ANOVA (confidence interval 0.05) adjusted using Bonferroni correlation was used for multi-variable analysis.

## Results and discussion

### Capture of *Cryptosporidium* in the micromixer microfluidic devices

**Fig. 1:**
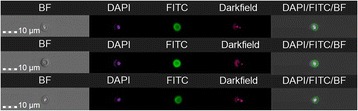
Cryptosporidium images from the different channels in the Imaging Flow Cytometer: Brightfield (BF), Darkfield, nuclear staining in DAPI and the Cryptosporidium specific Cry104 antibody (goat anti mouse FITC IgG secondary antibody), and the combined image. Images taken at 400× magnification and have been adjusted to enhance visual appearance

To investigate the effect of the velocity on the capture efficiency, 50 μl of *Cryptosporidium* oocyst solutions were initially flowed at a high concentration (1.5 × 10^6^oocysts / mL, ~ 75,000 oocysts) into the micromixers under 3 different flow rates. Microfluidic micromixers conjugated with BSA instead of the anti- *Cryptosporidium* antibody were used as controls. All experiments were performed in duplicate. IFC was used as a tool to quantitatively determine the capture efficiency of *Cryptosporidium* in the microfluidic micromixers. To this end, the unbound oocysts were recovered from the outlet of the microfluidic devices and prepared for analysis with IFC. Figure 1 shows *Cryptosporidium* oocysts recorded from different channels in the IFC: brightfield, DAPI, and Cry104 antibody labelled with a goat anti mouse FITC IgG secondary antibody, brightfield and darkfield.

**Fig. 2:**
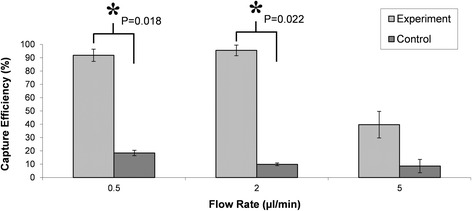
Capture efficiency of *Cryptosporidium* oocysts in the microfluidic devices at different flow rates calculated with Imaging Flow Cytometry. Light grey bars are the functionalized experimental results and the dark grey bars are the non-functionalized control devices. The average capture efficiency was 92%, 96% and 40% at 0.5, 2 and 5 μl/min respectively. Capture efficiency in the control devices was 18%, 10% and 9% for 0.5, 2 and 5 μL/min, respectively

Once the protocol to identify and count the oocysts with IFC was optimized, the capture efficiency for each flow rate was calculated. At 0.5 μL/min, the average capture efficiency was 92%, a maximum average capture efficiency of 96% was determined at 2 μL/min – only 3375 of the initial 75,000 oocysts were counted in the device’s waste (Fig. 2). There was statistical differences between bioconjugated and unconjugated control chip capture efficiencies for both 0.5 and 2 μl/min (students *t-test*, *P* < 0.05). There was, however, no difference in the capture efficiency between the 0.5 and 2 μl/min flow rates for the functionalised device. On the other hand, increased flow rates led to drastic decreases in the recovery yield with only 40% of the oocysts being recovered at 5 μl/min (45,225 in the waste). There was no statistical difference in capture between the 5 μl/min experiment and its respective control. This is due to the specific design of the F-shape micromixer, which maximizes mixing at lower flow rates. All control experiments resulted in a low non-specific capture of the oocysts within the non biofunctionalized micromixers (18%, 10% and 9% for 0.5, 2 and 5 μL/min, respectively).

**Fig. 3:**
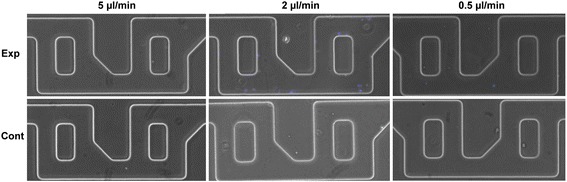
Microscopic images at 100× magnification of Cryptosporidium oocysts, stained in blue (DAPI), captured at the entrance of the micromixers, from PBS samples spiked with 1500 000 oocysts per ml

The use of IFC for characterization of the device performance is highly relevant, since it provides excellent quality of images for the micron-scale *Cryptosporidium* as well as the required high throughput quantification of the eluted samples. Standard flow cytometry, in contrast with IFC, encounters problems with the presence of auto fluorescent debris and clumps of *Cryptosporidium*, inhibiting proper identification and therefore, quantification [27]. The quantitative data obtained using IFC was confirmed using fluorescence microscopy (Fig. 3) of the micromixers. In good agreement with IFC, the highest number of *Cryptosporidium* oocysts retained in the micromixer was at the operating flow-rate of 2 μl/min. Minimal non-specific binding in control (non-functionalized) devices was also observed, confirming the specific nature of the binding. A flow rate of 2 μl/min was thus chosen to enhance throughput of the system without compromising capture efficiency.

**Fig. 4:**
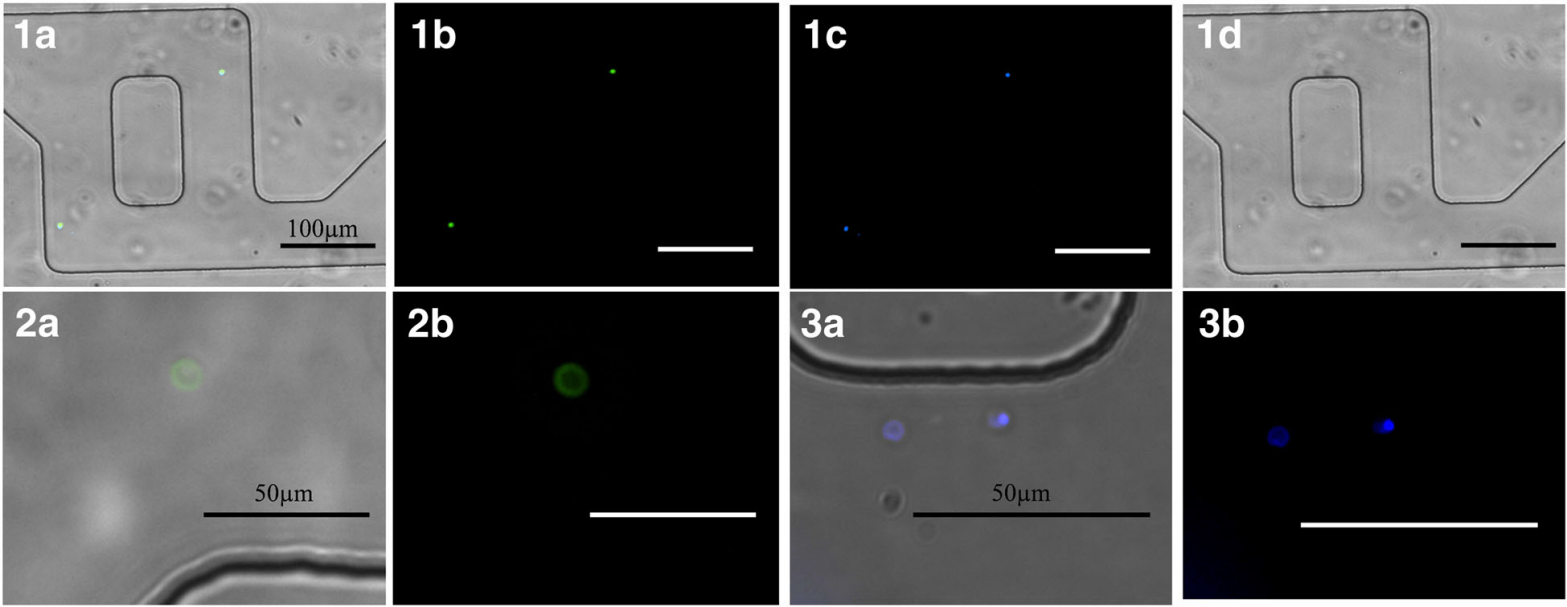
Microscopic images of the *Cryptosporidium* oocysts captured inside the functionalized microfluidic devices at the flow rate of 2 μl/min. *Cryptosporidium* oocysts are specifically recognized by Cry104 antibody, stained with a secondary FITC antibody and imaged at 100×, scale bar 100 μm (1). Composite image with brightfield (BF), FITC and DAPI channels (**1a**), DAPI channel (**1b**), FITC channel (**1c**) and BF image (**1d**). Images were also taken at higher magnification, 400×, scale bar 50 μm (**2**, **3**). Composite images with BF and FITC (**2a**) or BF and DAPI (**3a**), and individual FITC channel (**2b**), and DAPI channel (**3b**)

An important feature of the proposed microfluidic micromixer approach is the possibility to carry out high quality imaging directly within the devices themselves. Standard staining of the captured *Cryptosporidium* oocysts with the specific antibody Cry104 and DAPI, enabled straightforward visualization of the oocysts inside the micromixer as shown in Fig. 4.

**Fig. 5:**
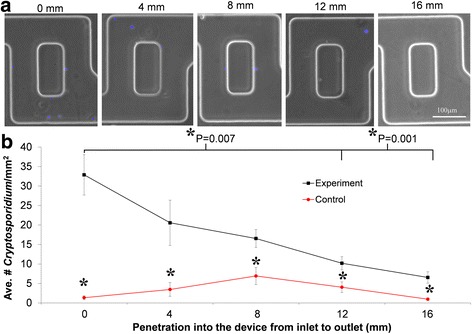
The surface density of *Cryptosporidium* oocysts in the microfluidic micromixers decreased from inlet to outlet. **a** Merged fluorescent microscopy images show the capture of oocysts in regions of the device at different distances from the inlet. (Left to right are distances of 0, 4, 8, and 12 mm from the inlet). **b** Average number of *Cryptosporidium* oocysts captured in a micromixer at 2 μl/min at different penetration lengths from inlet to outlet. The black line shows a functionalized experiment device and the red is the non-functionalized control device

The oocysts had a strong binding affinity to the antibody-functionalized micromixers as shown by preferential binding towards the inlet side (Fig. 5a). To confirm this observation, the number of oocysts captured at different lengths in the device was systematically counted using the following protocol: the number of oocysts captured was counted at 10 randomly chosen areas at different lengths into the device (0, 4, 8, 12 and 16 mm), 0 and 16 being the inlet and the outlet of the micromixer, respectively. Then the number of oocysts per area was averaged at each length and plotted in Fig. 5b. For each position there was statistically more oocysts captured in the Cry104 functionalised device than in the control (*p* < 0.05, students *t-test*). It was observed that the number of oocysts found in the microfluidic device is statistically different across the device decreasing with the penetration length with most binding occurring in the first part of the device (F statistic 0.0012, 1-way ANOVA). Specifically, at 0 mm penetration into the device there are statistically more *Cryptosporidium* than at 12 and 16 mm. For the control there was no difference in binding between any of the positions (F statistic 0.1039, 1-way ANOVA). This clearly demonstrates that *Cryptosporidium* oocysts have specific and strong binding affinity to the antibody bioconjugated device.

**Fig. 6:**
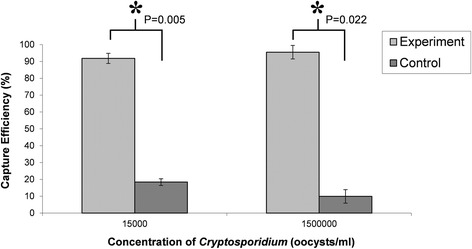
Dependence of the capture efficiency of *Cryptosporidium* oocysts in microfluidic micromixers at 2 μl/min at concentrations 15,000 and 1,500,000 crypto/ml, using imaging flow cytometry. Light grey bars are the functionalized experimental results and the dark grey bars are the non-functionalized control devices

To further confirm the validity of our system to isolate and quantify *Cryptosporidium* presence in water samples, the capture efficiency of *Cryptosporidium* oocysts was also studied at a lower concentration. For this purpose, 50 μl of two different concentrations of *Cryptosporidium*, 1.5 × 10^6^ and 1.5 × 10^4^ oocysts/ml (75,000 and 750 oocysts, respectively), were introduced at 2 μl/min, in the micromixers and the mean capture efficiencies were determined with IFC. As shown in Fig. 6, the capture efficiency remains constant independently of the *Cryptosporidium* concentration. For both, 75,000 and 750, there was a statistical difference between the functionalised device and the control (*p* < 0.05, students, *t-test*), with significally more binding in the functionalised device.

Once the ability of the system to capture and detect *Cryptosporidium* oocytes has been confirmed, for the system to be an analytical tool for quantification, a counting protocol needs to be defined. For this purpose, a calibration curve needs to be established to relate the number of oocytes counted by fluorescence microscopy to the real concentration of a given tested sample. Similarly to Fig. 5, we propose that the number of oocysts captured at different lengths in the device should be systematically counted at several randomly chosen areas. Then the average number of oocysts per area at different lengths could be plotted and fitted to a classical kinetics exponential decay, as in eq. 2:

2 $$ \boldsymbol{y}\left(\boldsymbol{x}\right)=\boldsymbol{A}{\boldsymbol{e}}^{-\boldsymbol{kx}} $$

where y(x) is the average number of oocytes in the device at a given length x. Following this protocol at different initial concentrations of *Cryptosporidium* and normalizing y(x), a calibration curve could be theoretically established for the dynamic range between the constant of decay k and the concentration of the solution. Alternatively, if the number of oocysts is very low, the total area of the microfluidic device can be directly scanned to find the concentration.

## Conclusion

The detection of pathogenic oocysts in water is an important health and environmental issue. A microfluidic micromixer device was developed to capture *Cryptosporidium* and enable direct in situ high quality microscopic observation. Imaging flow cytometry and fluorescence microscopy demonstrated that the oocysts had a strong binding affinity to the antibody-functionalized micromixers. Only minimal non-specific binding was observed in control devices, confirming the specific nature of the binding. A capture efficiency of 96% was determined under optimal conditions. Further studies are warranted beyond this proof of principle work to validate the proposed approach for its analytical potential for quantification in real samples. The main limitation of this microfluidic technology is its limited throughput, which restricts its application to water concentrates. However, it is anticipated that a minor redesign and/or multiplexing of the microfluidic mixers could lead to higher throughputs that would be readily compatible with volumes of water typically obtained from standard concentration methods as described in the EPA guideline. Besides its excellent efficiency, the main advantage of this technology is that its application would have strong potential to simplify the overall workflow, leading to notable time and resource savings. In summary, the microfluidic micromixer approach can capture *Cryptosporidium* in water concentrates and has the potential to accelerate and simplify the detection of *Cryptosporidium* and other microorganisms of concern in surface or drinking water.

## Abbreviations

APTMS: (3-aminopropyl) trimethoxysilane
BSA: Bovine Serum Albumin
DAPI: 4′,6-Diamidino-2-phenylindole dihydrochloride
ELISA: Enzyme-Linked Immunosorbent Assay
FBS: Fetal Bovine Serum
IFC: Imaging Flow Cytometer
MIC: Microfluidic Impedance Cytometry
PBS: Phosphate Buffered Saline
PDMS: Polydimethylsiloxane
